# Evidence for a Novel Mechanism Independent of Myocardial Iron in β-Thalassemia Cardiac Pathogenesis

**DOI:** 10.1371/journal.pone.0052128

**Published:** 2012-12-17

**Authors:** Ekatherina Stoyanova, Guy Cloutier, Hady Felfly, Wafaa Lemsaddek, Nicolas Ah-Son, Marie Trudel

**Affiliations:** 1 Institut de recherches cliniques de Montréal, Molecular Genetics and Development, Faculte de Medecine de L'Universite de Montreal, Montreal, Quebec, Canada; 2 Laboratory of Biorheology and Medical Ultrasonics, University of Montreal Hospital Research Center (CRCHUM), Montreal, Quebec, Canada; Mayo Clinic, United States of America

## Abstract

Human β-thalassemia major is one of the most prevalent genetic diseases characterized by decrease/absence of β-globin chain production with reduction of erythrocyte number. The main cause of death of treated β-thalassemia major patients with chronic blood transfusion is early cardiac complications that have been attributed to secondary iron overload despite optimal chelation. Herein, we investigated pathophysiological mechanisms of cardiovascular dysfunction in a severe murine model of β-thalassemia from 6 to 15-months of age in the absence of confounding effects related to transfusion. Our longitudinal echocardiography analysis showed that β-thalassemic mice first display a significant increase of cardiac output in response to limited oxygen-carrying erythrocytes that progressed rapidly to left ventricular hypertrophy and structural remodeling. Following this compensated hypertrophy, β-thalassemic mice developed age-dependent deterioration of left ventricular contractility and dysfunction that led toward decompensated heart failure. Consistently, murine β-thalassemic hearts histopathology revealed cardiac remodeling with increased interstitial fibrosis but virtual absence of myocardial iron deposits. Importantly, development of thalassemic cardiac hypertrophy and dysfunction independently of iron overload has uncoupled these cardiopathogenic processes. Altogether our study on β-thalassemia major hemoglobinopathy points to two successive phases resulting from severe chronic anemia and from secondarily induced mechanisms as pathophysiologic contributors to thalassemic cardiopathy.

## Introduction

β-thalassemia is among the most common monogenic hereditary disorder. β-thalassemia result from mutations that reduce or abolish expression of β-globin gene and thus hemoglobin production in red blood cells (RBC). According to the decrease in β-globin levels, two forms are distinguished: a moderate β-thalassemia intermedia and a severe β-thalassemia major. The hallmark of this disorder is hemolytic anemia with hypochromic and microcytic RBC. β-thalassemia are also characterized by marked destruction of RBC leading to stimulation of erythroid cell differentiation and elevated reticulocytes count. β-thalassemia major is a life-threatening condition that necessitates chronic blood transfusion therapy from early childhood whereas β-thalassemia intermedia is usually transfusion-independent.

β-thalassemia major causes high mortality and morbidity. In the 1970s, individuals with β-thalassemia major had a mean lifespan of ∼17 years [1] but under optimal iron chelation therapy in the last four decades, the prognosis has been greatly improved by >25 years [2]. β-thalassemia patients display severe systemic manifestations with damage to tissues or organs affecting mainly the spleen, liver, kidneys, lungs, bone marrow and heart [3]. Characteristic histopathologic anomalies in these various organs consist of iron deposits and/or fibrosis [4]. In addition, the spleen is affected by entrapment of RBC and by increased extramedullary hematopoiesis to compensate erythroid cell destruction. Most importantly, cardiac complications have been shown to be the most common cause of death in patients with β-thalassemia intermedia and major [5], [6].

Cardiac dysfunctions in β-thalassemia major have traditionally been attributed to iron-overload [7] related to repeated transfusions and increased intestinal absorption rate combined with a sustained state of increased cardiac output. It has been reported that the process of iron-related oxidative damage could lead to cardiac hypertrophy, cardiomyocyte degeneration and dysfunction associated with rhythm abnormalities [7], [8]. However, heart remodeling and failure is persistent even with optimal chelation in β-thalassemia major patients [9]. Unless heart in β-thalassemia major is highly prone to iron deposits in comparison to other organs, this raises the question of the existence of another etiology for cardiac complications. Furthermore, clinical studies in non-transfused β-thalassemia intermedia patients describe cardiac complications with no evidence of cardiac iron overload [10], [11]. Such clinical observation led us to hypothesize that β-thalassemia major cardiac dysfunction can occur in absence of transfusion related iron-overload and myocardial iron deposition.

Few mouse models of β-thalassemia have been developed and closely reproduced human β-thalassemia major or intermedia diseases. These models were generated to characterize the pathophysiology and to assess cell and gene therapy approaches. Among the murine models, only one reproduces β-thalassemia major (Hbb^d3(th)/d3(th)^), homo-βthal, and is the best-analyzed to date [12]. The homo-βthal mice exhibit typical severe β-thalassemia major phenotype with hematologic, histologic and physio-pathologic features including reduced fertility and death at early age [12], [13]. However, no study so far in any of the β-thalassemic mouse models has analyzed the functional and structural cardiac physiology.

To gain insights into the progressive cellular and molecular cardiac pathophysiology in β-thalassemia without the confounding effect of therapies, we have undertaken a longitudinal study from a cohort of untransfused homo-βthal mice from 6-months of age till death by non-invasive transthoracic echocardiography. These mice with virtual absence of cardiac iron deposition develop early alteration of left ventricular morphology followed by systolic dysfunction. Our results provide the first evidence that β-thalassemia major can induce iron-independent cardiac anomalies that likely progress to decompensation and lead to heart failure.

## Methods

### Mouse Strains

Experimental procedures were approved by the Animal Care Committee of the Clinical Research Institute of Montreal in accordance to the guidelines of the Canadian Council on Animal Care. Homozygous β-thalassemic (Hbb^d3(th)/d3(th)^) donor mice have a deletion of the murine β-globin major gene, in the globin diffuse haplotype, leaving only the β-minor gene intact [12]. These mice were backcrossed for >16 generations onto C57BL/6J and are considered on a homogeneous background. Congenic C57BL/6J-Gpi1^a^ donor and C57BL/6J-Gpi1^b^ recipient mice were differentiated with the marker glucose phosphate isomerase isotype (Gpi1) and were obtained from Jackson Laboratories (ME).

### Production of Bone Marrow Transplanted Mice

Bone marrow transplanted mice were produced as described previously [13]. Briefly, bone marrow cells were harvested from either homozygous β-thalassemic (homo-βthal: Hbb^d3(th)/d3(th)^) or wild-type C57BL6/J-Gpi1^a^ (Hbb^S/S^) donors. Two month-old male C57BL/6J recipients were exposed to a 8.75Gy lethal dose of irradiation (Mark I-68A-1 Irradiator, CA) and injected with 1.8×10^6^ hematopoietic cells from marrow of homo-βthal or of C57BL/6J-Gpi1^a^ controls (Hbb^S/S^). Bone marrow engraftment was evaluated in both mouse groups 2–5 months following transplantation and occasionally within the following year to verify sustained engraftment. Only recipients displaying complete hematopoietic engraftment were included in the study: recipients with the sole expression of either hemoglobin minor for homo-βthal mice (n = 46) or the specific glucose phosphate isomerase isotype marker *Gpi1^a^* for controls (n = 37).

### Hematological Analysis

Blood from transplanted homo-βthal and control mice (n = 8 from each group) as well as native homo-βthal (Hbb^d3(th)/d3(th)^) and control (n = 5 from both groups) was obtained from the submandibule and collected in tubes containing EDTA (Terumo Medical, Maryland). RBC and reticulocyte counts, hemoglobin (Hb) and hematocrit (Hct) levels, mean cellular volume (MCV) and mean cellular hemoglobin (MCH) were evaluated using an Advia 120 (Bayer, NY) with analysis software version 2.2.06 as previously [14]–[16].

### Echocardiography Examinations

Echocardiography was performed on male homo-βthal and control mice at 3 different ages: 6 months (n = 23 and 18), 10 months (n = 22 and 18), 14 months (n = 19 and 18) whereas in native homo-βthal (Hbb^d3(th)/d3(th)^; n = 3) analyzed at 14 months. Cardiac morphology and function were evaluated *in vivo* in anesthetized mice (0.0125 mL/g, Avertin 2.5%) by transthoracic echocardiography using an ultrasound biomicroscope Vevo770 (Visualsonics, Ontario) equipped with a 35 MHz probe. In addition, electrocardiogram (ECG) was monitored in all mice and body temperature maintained at 37±1°C using rectal thermometer (Indus Instruments, TX). Mean arterial blood pressure (MAP) was measured using tail-cuff monitoring system (XBP-1000, Kent Scientific, CT).

Left ventricle (LV) M-mode tracings were obtained at the level of papillary muscles using the two-dimensional parasternal long-axis view. Wall thickness and chamber dimensions measurements were averaged over 5 cardiac cycles and according to the American Society of Echocardiography guidelines [17]. End-diastolic (d) and end-systolic (s) left ventricle internal diameters (LVD), interventricular septum (IVS) and posterior wall (PW) thicknesses were measured. LV fractional shortening (FS) was calculated using the equation: FS(%) = [(LVDd−LVDs)/LVDd]×100. Ejection fraction (EF) was calculated as: [(LVEDV– LVESV)/LVEDV] based on LV end-diastole and end-systole volumes (LVEDV and LVESV). These were determined from Teichholtz’s formula [18]: LV volume = [7/(2.4+LVD)]×(LVD)^3^. LV mass was estimated using the equation [19]: LV mass = 1.055×[(IVSd+LVDd+PWd)^3^−(LVDd)^3^]. Relative wall thickness was assessed as the ratio between wall thickness and LV diameter: [(IVSd+PWd)/LVDd].

Aortic diameter (AoD) were measured in systole and diastole using M-mode tracings and averaged over 5 cardiac cycles. Doppler velocity waveforms were recorded in the aortic root and the velocity-time integral (VTI) was determined by semiautomatic analysis. VTI was averaged over 5 cardiac cycles. Cardiac output (CO) was calculated as follows [20]: CO = (AoD/2)^2^×π×VTI×HR, were HR was the heart rate obtained directly by ECG. Cardiac index (CI) was calculated by normalizing CO for body weight (BW) and expressed in milliliters per minute per gram of BW (mL⋅min^-1^⋅g^-1^).

Doppler ultrasound recordings were performed in the right common carotid artery 1–2 mm before the carotid bifurcation to measure peak systolic (S) and end-diastolic (D) velocities. Pourcelot index (PI), a commonly used parameter reflecting local vascular resistance to blood flow and vascular compliance was computed as previously [13]: PI = (S–D)/S and averaged over 10 cardiac cycles.

### Histopathological Examinations

Homo-βthal and control mice were sacrificed for pathologic analyses (n = 8 from each group) at 15 months and 7 months (n = 4 and 3, respectively). Heart, liver, kidney, spleen and lungs were excised and organ-to-BW ratios determined. Hearts, lungs, kidneys and spleens were fixed overnight in 10% phosphate-buffered formalin and paraffin-embedded. Tissue sections of 5 µm thickness were stained with hematoxylin-eosin, Prussian blue staining for iron analysis and Sirius red staining for interstitial fibrosis evaluation. Semi-quantitative levels of iron and fibrosis were evaluated from 8–10 photomicrographs per section at magnification 100X at same light intensity and exposition settings for each staining. Percentage of iron and collagen contents in each field was evaluated automatically using a computer assisted color threshold analysis using Matlab software (ver.7, MA).

### Statistical Analyses

Data are reported as means ± standard errors of the mean (SEM) over *n* observations, where *n* represents the number of mice per group. Longitudinal comparisons of cardiovascular parameters were assessed using a two-way ANOVA followed by a Student-Newman-Keuls test for multiple comparisons. Student’s unpaired *t*-test was used for comparison of hematological and histopathological parameters. All statistical analyses were performed using Sigma Stat (Systat, CA) and considered significant at *p*<0.05.

## Results

### Homo-βthal Mouse Model Phenotype

Homo-βthal mice deleted of both β-globin major genes were generated from bone marrow transplantation in order to obtain cohorts with several animals of same ages and same sex for longitudinal analysis. Complete engraftment of homo-βthal and control bone marrow in transplanted mice was determined from the hematologic glucose phosphate isomerase isotype marker and/or the sole presence of hemoglobin minor. To verify persistent and stable long-term engraftment, assessments were performed from 4 months of age onwards. Analysis of hematological profile was evaluated in adult mice to assess severity of β-thalassemia. The transplanted homo-βthal mice displayed features of β-thalassemia major with severe anemia as evidenced by markedly decreased RBC count, hemoglobin and hematocrit levels and consequently, exhibit decreased blood viscosity (Table 1). Additionally, mean cellular volume and mean cellular hemoglobin were significantly lower in homo-βthal mice (Table 1), correlating with erythrocyte microcytosis and hypochromia (data not shown). Severe reticulocytosis suggested increased erythropoiesis in homo-βthal mice in comparison to controls (Table 1). These hematologic parameters in transplanted homo-βthal mice are similar to those in native homo-βthal mice (Table S1).

**Table 1:** Hematologic parameters.

	Control	Homo-βthal
	(n = 8)	(n = 8)
**RBC (**10^6^/µL)	**8.2**±0.4	**4.8**±0.5^*^
**Hb (**g/dl)	**12.4**±0.6	**4.5**±0.5^†^
**Hct (**%)	**40.7**±1.4	**19.7**±1.7^†^
**MCV (**fl)	**49.7**±1.5	**41.8**±1.1
**MCH (**pg/RBC)	**15.0**±0.2	**10.4**±0.2^†^
**Retics (**%)	**6.6**±1.1	**31.2**±1.5^†^

Values are means±SEM. ^*^ *p*<0.01; ^†^ *p*<0.001 vs. control mice. RBC, red blood cell count; Hb, hemoglobin; Hct, hematocrit; MCV, mean RBC cellular volume; MCH, mean RBC cellular hemoglobin; Retics, Reticulocytes.

Consequent to complete engraftment of β-thalassemic hematopoietic stem cells, recipient mice showed significant decrease in lifespan expectancy relative to controls. Indeed, only 39.1% of transplanted homo-βthal mice were alive at 15 months whereas 91.9% of transplanted control mice were thriving at that age. Transplanted homo-βthal mouse model reproduced the typical hematological characteristics of severe β-thalassemia major [12] affecting their survival rate as reported in human β-thalassemia major [4].

### Distinct Histopathology in Homo-βthal Organs

**Figure 1:**
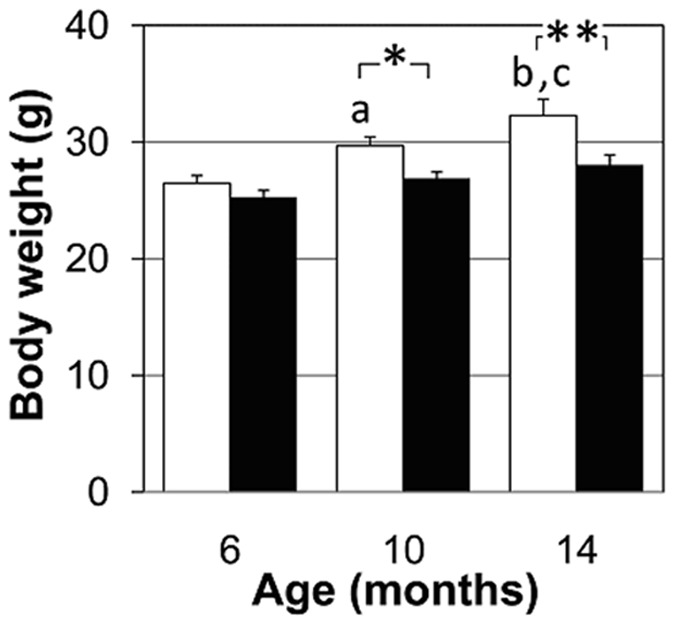
Longitudinal analysis of body weight in homo-βthal mice at 6, 10 and 14 months. Homo-βthal mice (filled bars) have significantly decreased body weight relative to controls (open bars) at 10 and 14 months (^*^ *p*<0.05, ^**^ *p*<0.01). Within the control group, body weight was increased at 10 and 14 months vs 6 months (^a^ *p*<0.05, ^b^ *p*<0.001), and 14 months vs 10 months (^c^ *p*<0.05). Values are means±SEM and analyzed by two-way ANOVA.

Since β-thalassemia patients have hampered developmental growth, we monitored body weight (BW) in homo-βthal mice from 6 months of age (Figure 1). At 6 months, BW was slightly reduced by ∼5% in homo-βthal mice compared to control mice (p = 0.2). The decrease in BW of homo-βthal mice dwindled further at 10 and 14 months of age by 10 and 14%, respectively in comparison to age-matched controls. These findings of significant growth impairment indicate a general physiological impact of the disease in homo-βthal mice from 10 months of age onwards.

Table 2 shows organ-to-BW ratios in both thalassemic and control groups at ∼15 months of age. All organ-to-BW ratios were significantly higher (*p*<0.05) in the target group and were comparable to those of native homo-βthal mice (data not shown). As expected, the most notable organ enlargement was the spleen by ∼4.8-fold, a typical characteristic of severe murine β-thalassemia [15]. While lungs- and liver-to-BW ratios were increased by 1.2- to 1.3-fold respectively, the heart- and kidney-to-BW ratios were markedly increased by 1.6- to 1.7-fold in homo-βthal mice (Table 2).

**Table 2:** Organ-to-body ratios of homo-βthal mice.

		Organ-to-body weight ratio (mg/g)				
	n	Heart	Liver	Spleen	Lungs	Kidney
**Control**	8	**5.5**±0.3	**45.0**±1.2	**2.5**±0.2	**6.2**±0.3	**6.6**±0.3
**Homo-βthal**	8	**8.8**±0.5^‡^	**54.2**±3.6^*^	**12.1**±1.1^‡^	**8.3**±0.7*	**11.7**±1.5^†^

Organ-to-body weight ratios in 15 month-old control and homo-βthal mice.

Values are means±SEM. ^*^ *p*<0.05; ^†^ *p*<0.01; ^‡^ *p*<0.001 vs. control mice.

**Figure 2:**
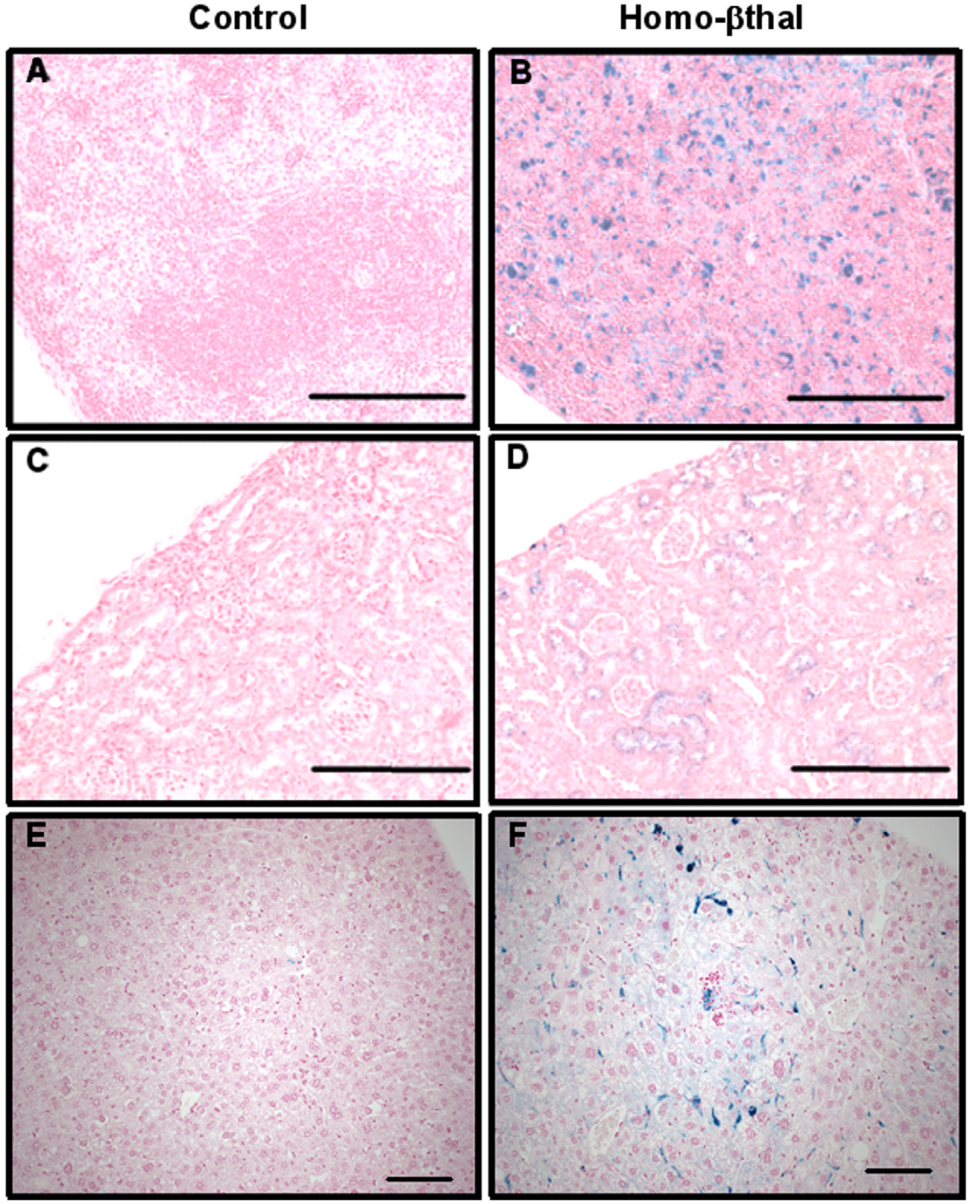
Histopathologic analysis of iron deposition in 15-month mouse thalassemic tissues. Tissue sections were stained for iron deposits with Prussian blue and counterstained with nuclear fast red. A) Spleen of control mice with well-organized white and red pulp displayed mild iron deposits B) Spleen of homo-βthal showed numerous iron deposits throughout the parenchyma. C) Kidney of control mice did not exhibit presence of iron. D) Kidney of homo-βthal mice revealed iron deposits in proximal tubular cells of the cortical region. E) Liver of control mice did not exhibit presence of iron. F) Liver of homo-βthal mice revealed iron deposits in Kupffer cells and in parenchymal cells. (Magnification×20; A-D: bars, 200 µm; E,F: bars, 100 µm).

**Figure 3:**
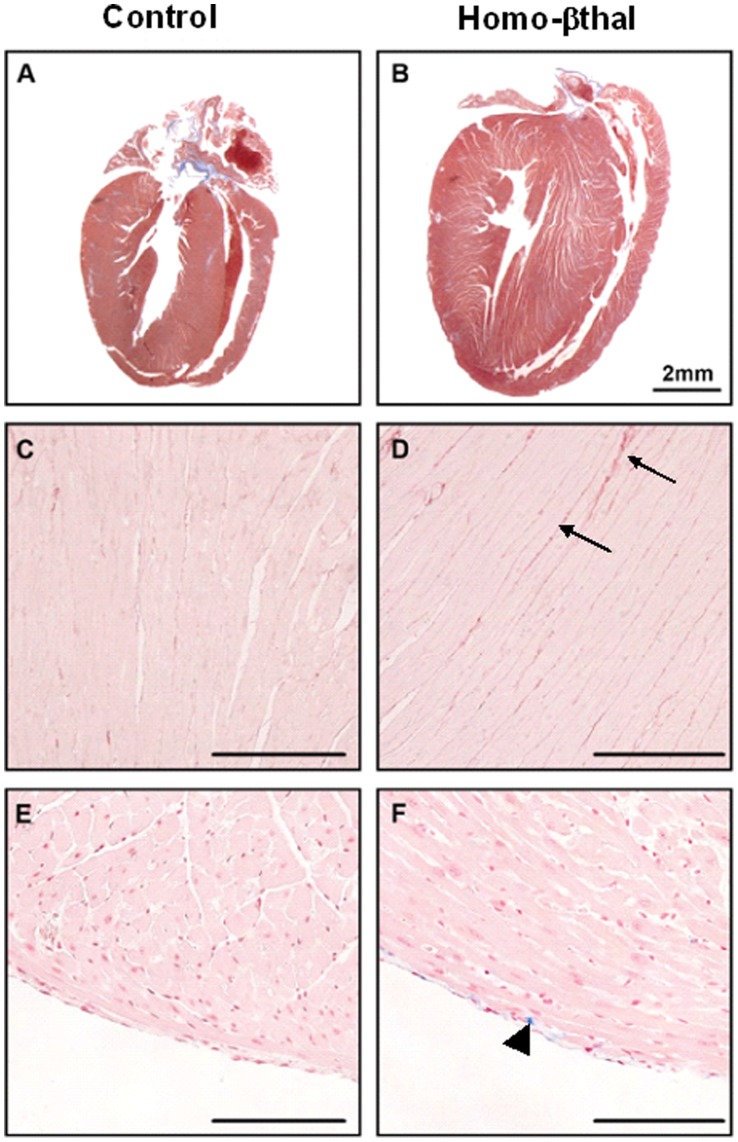
Macroscopic and microscopic alterations of murine thalassemic hearts. Representative cardiac macroscopic phenotype of 15-months control (A) and homo-βthal (B) show important heart hypertrophy in homo-βthal mice; hematoxylin-eosin stain. Heart tissue sections of control (C) in comparison to homo-βthal (D) stained with Sirius red revealed markedly enhanced collagen levels (arrow) in homo-βthal hearts. Histological cardiac analysis of control (E) and homo-βthal (F) sections were comparable with occasional detectable iron signals (arrowhead); Prussian blue stain and nuclear fast red counterstain. (Magnification×40; Bars, 100 µm).

As an initial assessment in thalassemic and control groups, levels of serum iron concentration were monitored. Evidence of iron overload was provided by significantly higher plasma iron levels in homo-βthal mice (39.1±4.0 umol/l; n = 7) than controls (27.5±3.1 umol/l; n = 8; p<0.04). Histopathological semi-quantitative analysis of heart, spleen, lungs, liver and kidney were performed including specific stainings, Prussian blue for iron and Sirius red for interstitial fibrosis (Table 3). Presence of abundant iron in the spleen is a typical feature of severe β-thalassemia [15] secondary to reticuloendothelial RBC destruction and extramedullary hematopoiesis (Figure 2B, Table 3). Accordingly, important splenic collagen deposition was measured in homo-βthal mice, 1.9-fold above those of controls (Table 3, Figure S1). In the lungs of homo-βthal mice, a significant 4-fold increase of iron deposits was quantified throughout the tissue in comparison to very low to undetectable levels in controls (*p*<0.05) (Table 3). However, no increase in interstitial fibrosis was detected in the lungs of homo-β-thal mice. Similarly to the lungs, the kidneys of homo-βthal mice displayed no interstitial fibrosis but had high levels of iron deposits, mainly localized to the cortical region (Figure 2D, Table 3). The presence of iron in proximal tubular cells may be indicative of free hemoglobin renal reabsorption due to chronic hemolysis. Tissue iron deposits were also quite abundant in liver of homo-βthal mice relative to controls (Figure 2E, 2F). As illustrated in Figure 3A and 3B, homo-βthal mice exhibited marked cardiac hypertrophy. Importantly, this enlargement was associated with a 1.7-fold increase in interstitial collagen deposition diffusely distributed throughout the myocardium when compared to controls (*p*<0.001, Figs. 3C and 3D). This increase in cardiac collagen content was characteristic of the 15 month-old homo-βthal mice (Table 3) since 7 month-old homo-βthal mice exhibited similar low levels of fibrosis (2.6±0.5%; n = 4) as in controls (2.6±1.0%; n = 3) (Figure S2). In addition, iron deposits in the hearts of homo-βthal mice were extremely sparse in contrast to all other organs. When detected, these deposits in homo-βthal hearts were located in focal region of the outer layer of the myocardium or epicardium (Figure 3F, Figure S2), were non-significant (*p*>0.05) and comparable to those of controls (Figure 3E) and to wild-type C57Bl/6J mice of same genetic background (0.12±0.11%; n = 9) (H.F. and M.T., 2007). These results indicate that cardiac hypertrophy is not directly linked to iron deposits but associated with progressive fibrosis at later age in thalassemia.

**Table 3:** Histopathologic assessment of homo-βthal mice.

	Iron deposits (%)			Collagen content (%)		
Organ	Control	Homo-βthal	Hbbd3(th)/d3(th)	Control	Homo-βthal	Hbbd3(th)/d3(th)
	(n = 5)	(n = 5)	(n = 3)	(n = 5)	(n = 5)	(n = 3)
**Heart**	**0.25**±0.19	**0.12**±0.03	**0.21**±0.11	**4.0**±0.3	**6.9**±0.3^‡^	**8.0**±2.2
**Spleen**	**3.60**±1.46	**17.68**±4.14^*^	**10.11**±1.92	**3.6**±0.6	**7.0**±1.2^*^	**9.0**±2.0
**Lungs**	**0.20**±0.05	**0.81**±0.22^*^	**0.66**±0.12	**5.4**±0.6	**4.4**±1.0	**4.2**±0.2
**Kidney**	**0.54**±0.50	**4.47**±1.46^*^	**3.8**±1.4	**8.0**±1.3	**7.4**±1.1	**9.4**±0.6
**Liver**	**0.17**±0.05	**1.25**±0.22^‡^	**0.53**±0.07^‡^	**4.43**±0.60	**10.41**±1.77^**^	**2.33**±0.22

Semiquantification of iron deposits and collagen content in 15 month-old control, homo-βthal and native Hbb^d3(th)/d3(th)^ mice.

Values are means±SEM. ^*^ *p*<0.05; ^**^ *p*<0.002; ^†^ *p*<0.01; ^‡^ *p*<0.001 vs. control mice.

### Early Onset of Left Ventricle Structural Alterations in Homo-βthal Mice

**Figure 4:**
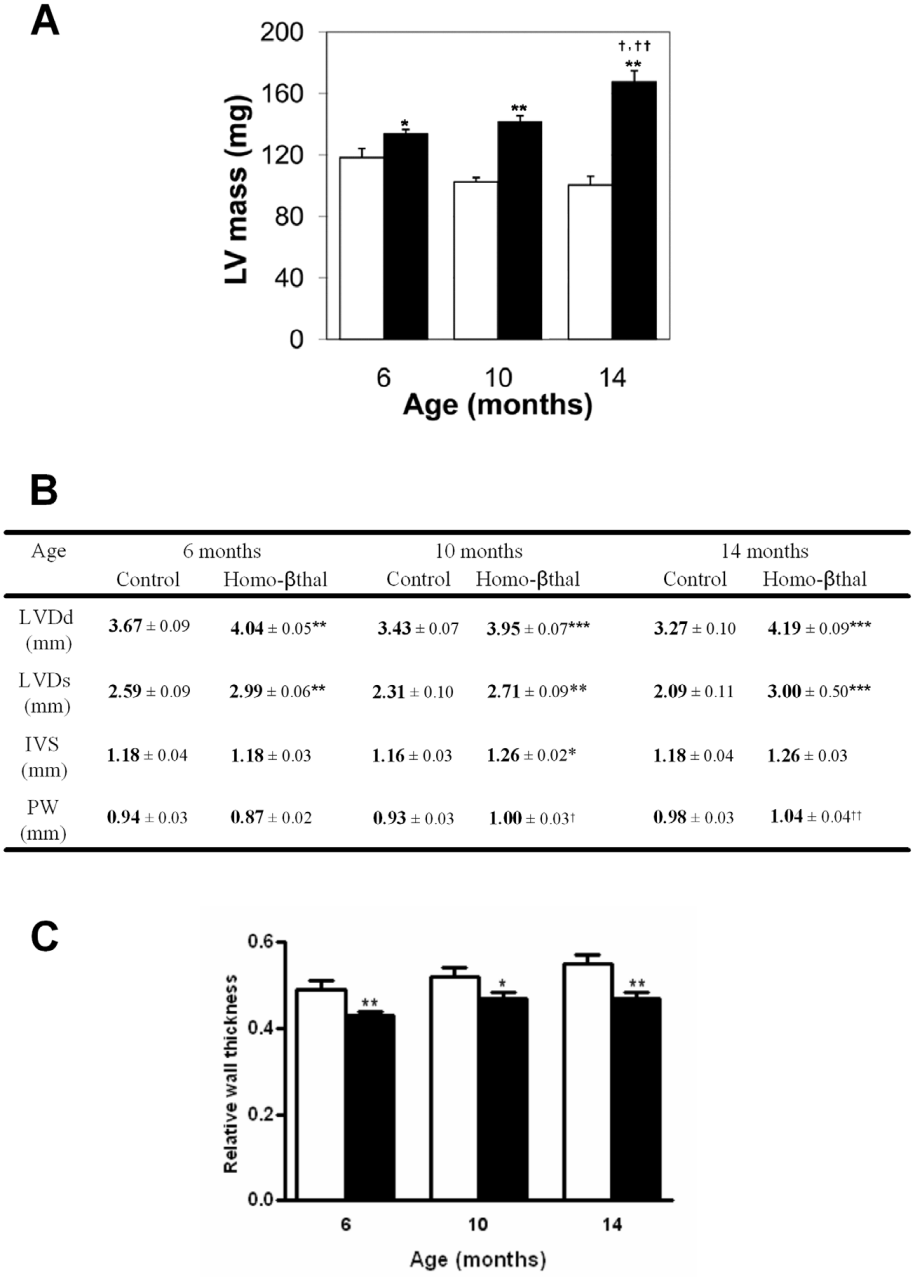
Longitudinal cardiac morphologic analysis of homo-βthal mice. A) Longitudinal evaluation of LV mass by echocardiography in control (open bars) and homo-βthal mice (filled bars) at ages 6, 10 and 14 months show a progressive increase of LV mass in homo-βthal mice with age. Values are means±SEM. Comparison of LV mass between homo-βthal and control mice (^*^ *p*<0.05, and ^**^ *p*<0.001) and within homo-βthal mouse groups at 10 vs14 months (^†^ *p*<0.01) and at 6 vs 14 months (^† †^ *p*<0.001). B) Comparison of echocardiographic measurements at ages 6, 10 and 14 months in homo-βthal (n = 23, 22, 19) and control (n = 18) mice. Cardiac parameters consist of LVDd, left ventricule diameter in diastole; LVDs, left ventricule diameter in systole; IVS, interventricular septum; PW, posterior wall. Values are means±SEM. Cardiac parameters were significantly increased in homo-βthal mice compared to same age controls (^*^ *p*<0.05, ^**^ *p*<0.01 and ^***^ *p*<0.001) and within the homo-βthal group, cardiac parameters were increased at 10 and 14 months vs 6 months (^†^ *p*<0.05 and ^††^ *p*<0.01). C) Relative wall thickness of homo-βthal (filled bars) relative to control (open bars) mice was consistently diminished at the different analyzed ages 6, 10 and 14 months (^*^ *p*<0.05 ^**^ *p*<0.01).

In addition to the molecular cardiac changes identified in homo-βthal mice by histopathological analysis, we investigated cardiac morphology and function by echocardiography. Since complete engraftment was observed at 5 months, our longitudinal echocardiography analyses were initiated from 6 months of age. Left ventricle (LV) mass estimation and dimension measurements were assessed by M-mode tracings (Figures 4A, 4B and Figure S3). At 6 months, homo-βthal mice revealed a mild LV mass increase of 13% compared to controls (*p*<0.05), indicating early development of LV hypertrophy. Despite no BW increase in homo-βthal mice, LV mass was significantly and progressively increased in 10 and 14 month-old mice by 38% and 67%, respectively (Figure 4A). This LV mass increase was similar to the 56% increase at 14-month old in native homo-βthal mice (n = 3). Control mice displayed similar LV mass at 10 and 14 months with a slight (∼13%) decrease compared to 6 months. Consistent with the LV mass increase in homo-βthal mice, detailed echocardiographic measurements showed an important and significant increase of LV diameters in both systole (LVDs) and diastole (LVDd) at all ages (Figure 4B and Figure S3). In addition, interventricular septum (IVS) and posterior wall (PW) thicknesses tended to increase in homo-βthal mice at 10 and 14 months of age, further supporting age-dependent progression of LV hypertrophy. To evaluate geometric pattern of LV chamber dilation and wall thickening, we calculated relative wall thickness with respect to LV cavity diameter (Figure 4C). Compared to controls, relative wall thickness was decreased in homo-βthal mice at all ages (∼12 to ∼9%), thereby characterizing the LV remodeling pattern as an eccentric hypertrophy.

### Progressive Cardiovascular Impairment in Homo-βthal Mice

**Figure 5:**
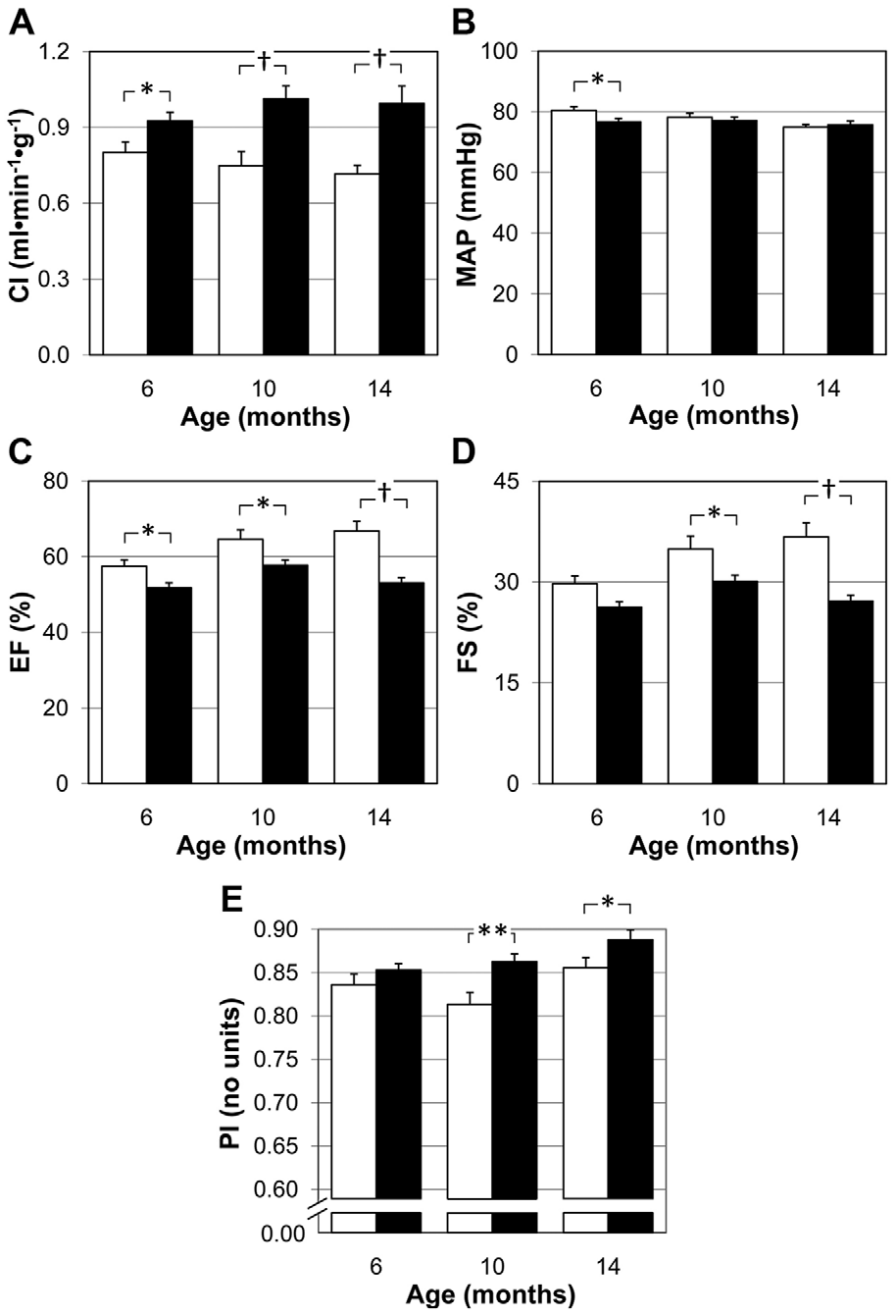
Impaired cardiac function in homo-βthal mice. A) Longitudinal measurements of cardiac index (CI) in homo-βthal mice (filled bars) at ages 6, 10 and 14 months relative to control (open bars) was increased. B) Mean arterial pressure (MAP) evaluated by tail-cuff approach was comparable in control (open bars) and homo-βthal mice (filled bars). C) Left ventricular ejection fraction (EF) in homo-βthal mice (filled bars) decreased relative to control (open bars) at all ages. D) Left ventricular fractional shortening (FS) in homo-βthal mice (filled bars) declined at 10 and 14 months of age compare to control (open bars). E) Doppler Pourcelot indices (PI, no units) of the common carotid artery in 6, 10 and 14 month-old control (open bars) and homo-βthal mice (filled bars) indicated altered vascular hemodynamics in the homo-βthal mice. Values are means±SEM.^ *^ *p*<0.05, ^**^ *p*<0.01 and ^†^ *p*<0.001 vs. same age control mice.

To investigate whether the cardiac hypertrophic alterations in homo-βthal mice were associated with functional impairment, we evaluated cardiac index (CI) by normalizing the cardiac output to BW and LV contractile function. The longitudinal CI analysis of control mice displayed a mild but non-significant decrease. In contrast, CI in homo-βthal mice were significantly increased by 16% (*p*<0.05), 35% (*p*<0.001) and 39% (*p*<0.001) at 6, 10 and 14 months, respectively, when compared with age-matched controls (Figure 5A).

Cardiac function was evaluated by LV ejection fraction (EF) and LV fractional shortening (FS) calculations (Figures 5C and 5D). EF was decreased in all age groups of homo-βthal mice and declined further with age, up to 20% loss relative to controls. FS seemed unaffected in 6 month-old homo-βthal mice, but was considerably decreased by 10 months of age compared with age-matched controls. The decrease in EF and FS was comparable to that of native homo-βthal mice (n = 3) by 12 and 14% respectively at ∼15 month of age. These results indicated the development of a progressive left ventricle contractile dysfunction in homo-βthal mice without evidence of hemosiderosis.

As shown in Figure 5B, mean arterial blood pressure (MAP) was slightly lower (*p*<0.05) in 6 month-old homo-βthal mice when compared with age-matched controls. At all other ages no difference in MAP was observed. In addition, local common carotid vascular hemodynamics was monitored to have insight into vascular compliance and resistance to blood flow by using the Pourcelot resistive index (PI). As shown in Figure 5E, homo-βthal mice displayed significantly higher PI at 10 (*p*<0.01) and 14 (*p*<0.05) months compared to age-matched controls, suggesting impaired carotid vascular hemodynamics with increased arterial resistance.

## Discussion

Our longitudinal and systematic study of thalassemia heart pathophysiology provides evidence of novel determinants for progression of cardiac structure remodeling and dysfunction leading to insufficiency. Herein, we demonstrate for the first time that untreated β-thalassemia major mice display severe cardiac complications. In addition to compensated left ventricular dilation and hypertrophy, our thalassemic homo-βthal mouse model develops sequentially progressive contractile functional impairment with decreased LV fractional shortenings. Importantly, this process was associated with elevated interstitial fibrosis but occurred independently of myocardial iron loading. Since our homo-βthal mouse dissociated the thalassemic cardiopathy from presence of myocardial iron, these findings argue for the existence of distinct mechanism(s) that are major contributors to thalassemic cardiac pathogenesis.

Characterization of the primary events occurring in β-thalassemia major cardiac pathogenesis from our mouse model revealed early onset of morphologic adverse changes. In response to reduced oxygen-carrying potential of β-thalassemic erythrocytes and tissue hypoxia in engrafted homo-βthal mice, a compensatory mechanism is induced by increasing the cardiac index or output. This normal hemodynamic adaptation mechanism results from elevated cardiac preload and venous return and decreased afterload via reduced blood viscosity and blood pressure as detected in homo-βthal mice early on [21], [22]. Within 4 months of exposure to progressive anemia, the homo-βthal mice developed a rapidly enhanced LV mass and decreased BW comparable to that of the 12-weeks anemia-induced iron deficiency in rat [23]. In parallel, a gradual increase in LV diameter at diastole and systole in homo-βthal mice showed early dilatation consistent with a sustained anemic state [24]–[26]. These morphologic alterations lead in the initial phase to a typical eccentric LV pattern of hypertrophy in the homo-βthal mice. Interestingly, anemia-induced cardiomegaly has also been reported for other vertebrates including a genetically-induced mutant in Zebrafish [27]. A primary event in homo-βthal mice is early cardiac morphologic remodeling presumably as a compensatory mechanism for the anemia and/or ischemia. This physiologic mechanism should lead to the apparent correction of the hypoxic state by stationary increased cardiac index.

In contrast to findings in chronic anemia states [24], [28], [29], the CI in homo-βthal mice rose further and reached a maximum at 10 months of age. Moreover, a persistent increase in LV mass and diameter demonstrate LV remodeling throughout life with progressive deterioration of the homo-βthal cardiac geometry/structure. From 10 months of age, the homo-βthal mice displayed a combined pattern of eccentric LV hypertrophy and of wall thickening. Such cardiac hypertrophy evolved to cardiac dysfunction in homo-βthal mice, with gradual alteration and decline of LV function. The mild decrease in cardiac function, as defined by FS and EF, at 6 months with no major impact in hemodynamic function indicated that LV hypertrophy in homo-βthal mice preceded cardiovascular functional alterations. Notably by 10 months of age and onwards, significant degeneration of cardiac function suggested that additional or secondary factors or events are triggered at later stage. An important contributor to the thalassemic pathophysiology of cardiac dysfunction is likely the impaired circulatory flow hemodynamics as indicated by the mild but significant increase in carotid resistance from the Pourcelot index despite decreased blood viscosity. This process may arise from vascular endothelial dysfunction and structural wall remodeling. In addition, increased ventricular stress caused by cardiac dilatation from anemia-induced conditions similar to homo-βthal mice was associated with myocyte hypertrophy and hyperplasia [25]. Long-term exposure to ventricular stress is likely to exert adverse cellular and molecular effect as evidenced in later stage by presence of substantial fibrosis in the myocardium of homo-βthal. Consequently, this interstitial fibrosis is expected to impact on ventricular contractility and cause myocardial stiffness [30]. Importantly, our characterization of homo-βthal mice showed severe myocardial dysfunction despite virtual absence of cardiac iron.

Our study uncovered the existence of a novel mechanism(s) critical in β-thalassemia cardiac dysfunction independently from myocardial iron loading. Until now, iron is considered the main factor responsible for cardiac failure in the chronically transfused human β-thalassemia major [4], [7]. Consistently, lifespan in thalassemic patients improved significantly upon iron-chelation therapy [8], [31]. Nevertheless, cardiac dysfunction was reported in some thalassemic patients under chelation and it was assumed to result from low therapy compliance [32] and/or from differential heart and liver iron-loading kinetics [33], [34]. Impaired cardiac function in these thalassemic patients supports our data of additional mechanism(s) distinct from myocardial iron leading to cardiac failure and also raises questions on iron assessment. In fact, standard analysis of iron or ferritin levels in serum of patients do not adequately reflect differential iron storage levels in different organs and are limited by low reproducibility [35]. While only direct tissue biopsy can quantify cardiac iron rigorously, the non-invasive nuclear magnetic resonance relaxometry is sensitive to detect iron in tissues [36], [37] with 80% reliability in comparison to heart biopsy [38] and the T2* approach is now favored to evaluate chelator efficacy in patients [39]. Analogous to the human pattern of iron organ distribution, homo-βthal mice had excess iron in vital organs such as spleen, lungs and kidney, probably originating from increased gastrointestinal absorption. Despite increased plasma iron levels, absence of iron in the heart of homo-βthal mice highlighted the difference between organs for uptake and storage of iron. This difference may reflect expression specificity of transferrin receptor levels that are much lower in the heart than in other organs [40], [41]. Hence, our findings show that development of LV hypertrophy and the later onset of LV dysfunction in homo-βthal mice occurs independently of myocardial iron deposition. This murine thalassemic iron-independent LV dysfunction is also consistent with the cardiac dysfunction reported in untransfused thalassemia intermedia [11] and in occasional thalassemic major transfused patients without cardiac siderosis [9]. Hence, a distinct mechanism(s) is most likely responsible for the cardiac dysfunction in these non-transfused homo-βthal mice. Our results show that additional critical determinant(s) are at the basis of the thalassemic cardiac pathophysiology and argue that iron is a contributor and likely a precipitating factor in human cardiac dysfunction.

An important finding from this longitudinal analysis is the unraveling of a novel pathophysiologic mechanism independent of direct myocardial iron deposition that leads to cardiac complications in β-thalassemia major and possibly, in β-thalassemia intermedia. Our results show that the thalassemic heart primarily adapts by increasing cardiac output state as in all anemias. The chronic anemic state of thalassemia caused morphologic alterations including LV dilation and hypertrophy. In thalassemia however, a secondary response is induced: the cardiac function progressively deteriorates to LV decompensation, dysfunction and eventually heart failure. This process can result from the contribution of various factors successively or cooperatively such as vascular endothelial dysfunction and vasculopathy as described in sickle cell disease another hemolytic condition [42]–[45] or as compensated LV structural remodeling itself and/or loss of contractile reserve due to myocardial stiffness (as indicated herein). Most importantly, studies should be directed at characterization of the molecular pathophysiologic determinants of thalassemic cardiac pathogenesis. While major efforts for optimization of chelation therapy are essential for β-thalassemia, our data predict premature cardiac morbidity and mortality in thalassemic patients despite strict lifelong optimal chelation therapy.

In summary, our study on β-thalassemic mouse models is the first to show a cardiopathological mechanism with atypical fibrosis that is independent of iron overload. These findings are likely to revolutionize our cardiac β-thalassemia biomedical basic concepts and influence clinical practice. Our β-thalassemia model also provides a mean to investigate the molecular determinants responsible for pathophysiologic structural and functional cardiac anomalies and for assessment of innovative therapeutic interventions that could be pertinent for RBC disorders at large and even other cardiopathologies.

## Supporting Information

Figure S1 **Histopathologic analysis of interstitial fibrosis in 15-month mouse thalassemic tissues.** Spleen of control mice (A) stained with Sirius red has scattered and mild fibrosis whereas the spleen of homo-βthal mice (B) show elevated levels of fibrosis (arrow) and presence of unstained iron (star). Kidneys of control (C) and homo-βthal (D) mice show no difference in level of fibrosis.
(TIF)

Figure S2 **Histopathologic analysis of iron deposits and interstitial fibrosis in 10-month mouse thalassemic tissues.** A, B) Hearts from both control mice and of homo-βthal mice at ∼10 months of age stained with Sirius red do not exhibit presence of iron deposits. C, D) In comparison to the control heart, heart of the homo-βthal mice at ∼10 months of age displayed mild levels of fibrosis. E, F) Hearts from both control and of homo-βthal mice at ∼7months of age have indistinguishable levels of fibrosis.
(TIF)

Figure S3 **Transthoracic M-mode tracings of the left ventricle.** Representative tracings in 14 month-old control (left) and homo-βthal (right) mice. LVD is the left ventricular diameter in diastole (white line).
(TIF)

Table S1 **Hematologic parameters in C57Bl6J and Hbb^d3(th)/d3(th)^ mice.** RBC, red blood cell count; Hb, hemoglobin; Hct, hematocrit; MCV, mean RBC cellular volume; MCH, mean RBC cellular hemoglobin; Retics, Reticulocytes.
(TIF)
