# Supplementary figures and images for: Evidence for a Novel Mechanism Independent of Myocardial Iron in β-Thalassemia Cardiac Pathogenesis
Source: PLoS One. 2012 Dec 17;7(12):e52128. doi: 10.1371/journal.pone.0052128 (PMC3524169; doi:10.1371/journal.pone.0052128)

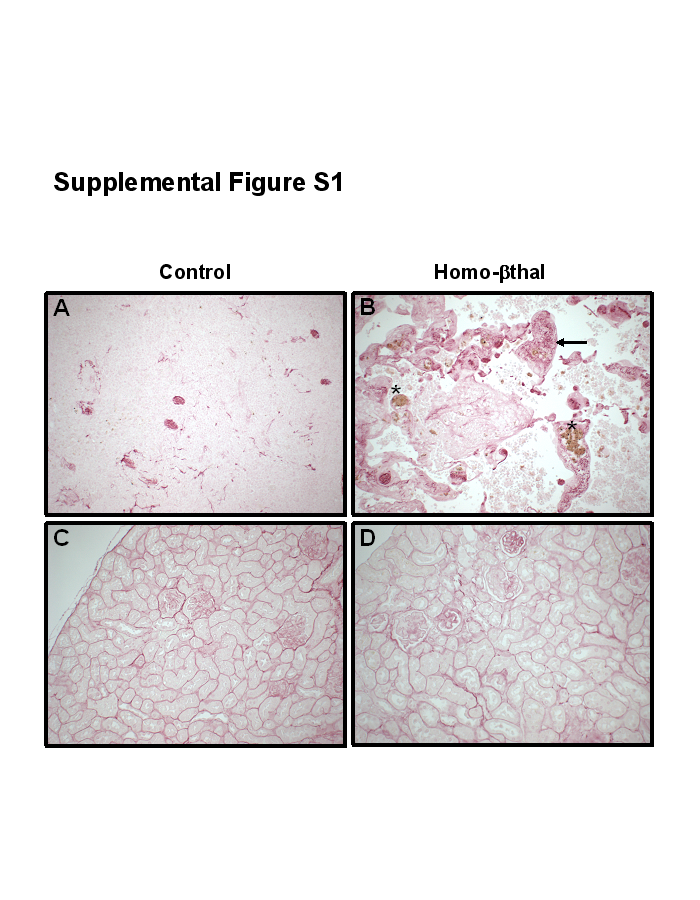

Supplement: Figure S1 — Histopathologic analysis of interstitial fibrosis in 15-month mouse thalassemic tissues. Spleen of control mice (A) stained with Sirius red has scattered and mild fibrosis whereas the spleen of homo-βthal mice (B) show elevated levels of fibrosis (arrow) and presence of unstained iron (star). Kidneys of control (C) and homo-βthal (D) mice show no difference in level of fibrosis. (TIF) [file pone.0052128.s001.tif]

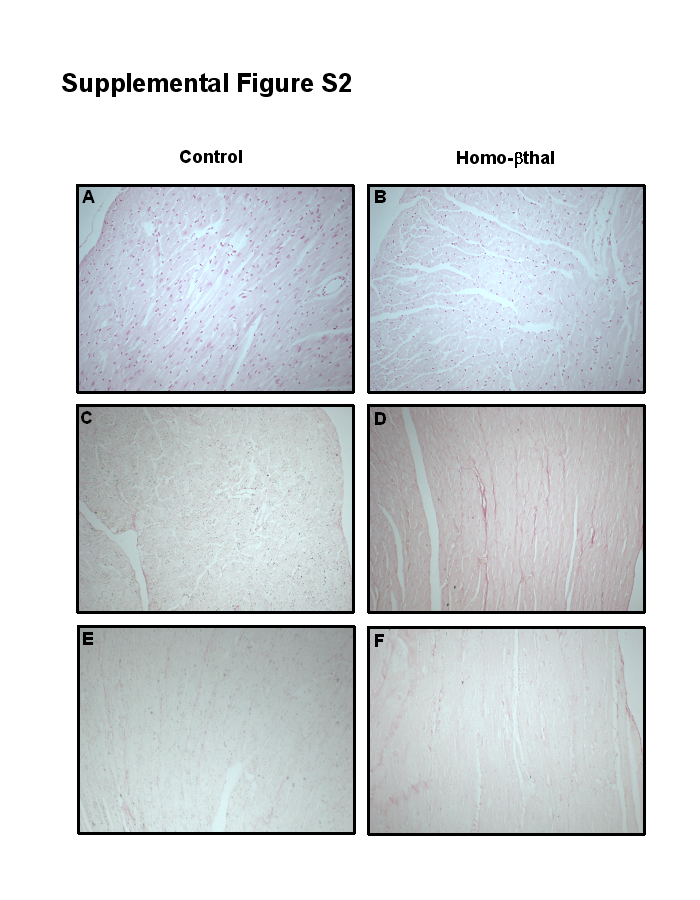

Supplement: Figure S2 — Histopathologic analysis of iron deposits and interstitial fibrosis in 10-month mouse thalassemic tissues. A, B) Hearts from both control mice and of homo-βthal mice at ∼10 months of age stained with Sirius red do not exhibit presence of iron deposits. C, D) In comparison to the control heart, heart of the homo-βthal mice at ∼10 months of age displayed mild levels of fibrosis. E, F) Hearts from both control and of homo-βthal mice at ∼7months of age have indistinguishable levels of fibrosis. (TIF) [file pone.0052128.s002.tif]

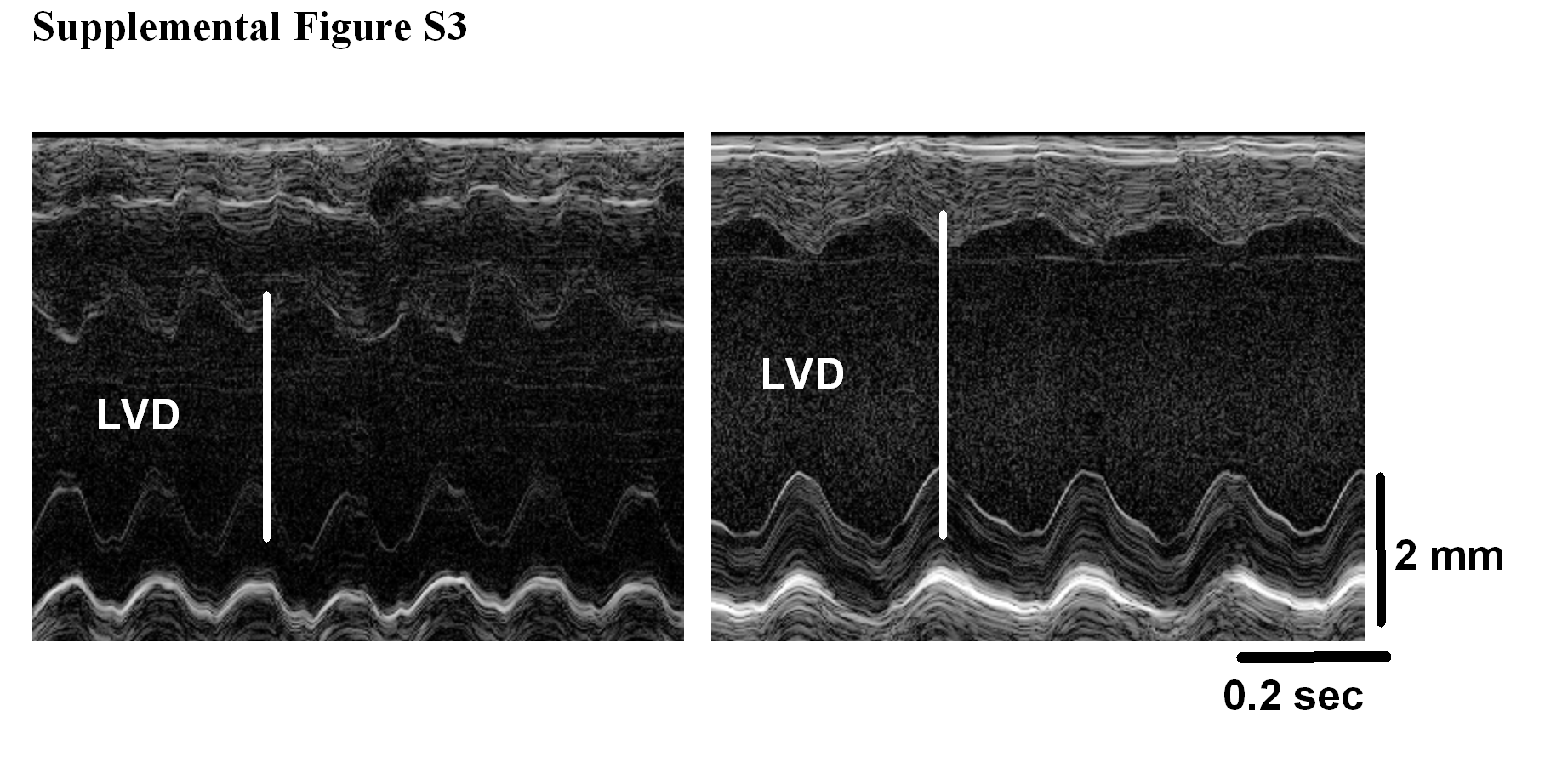

Supplement: Figure S3 — Transthoracic M-mode tracings of the left ventricle. Representative tracings in 14 month-old control (left) and homo-βthal (right) mice. LVD is the left ventricular diameter in diastole (white line). (TIF) [file pone.0052128.s003.tif]

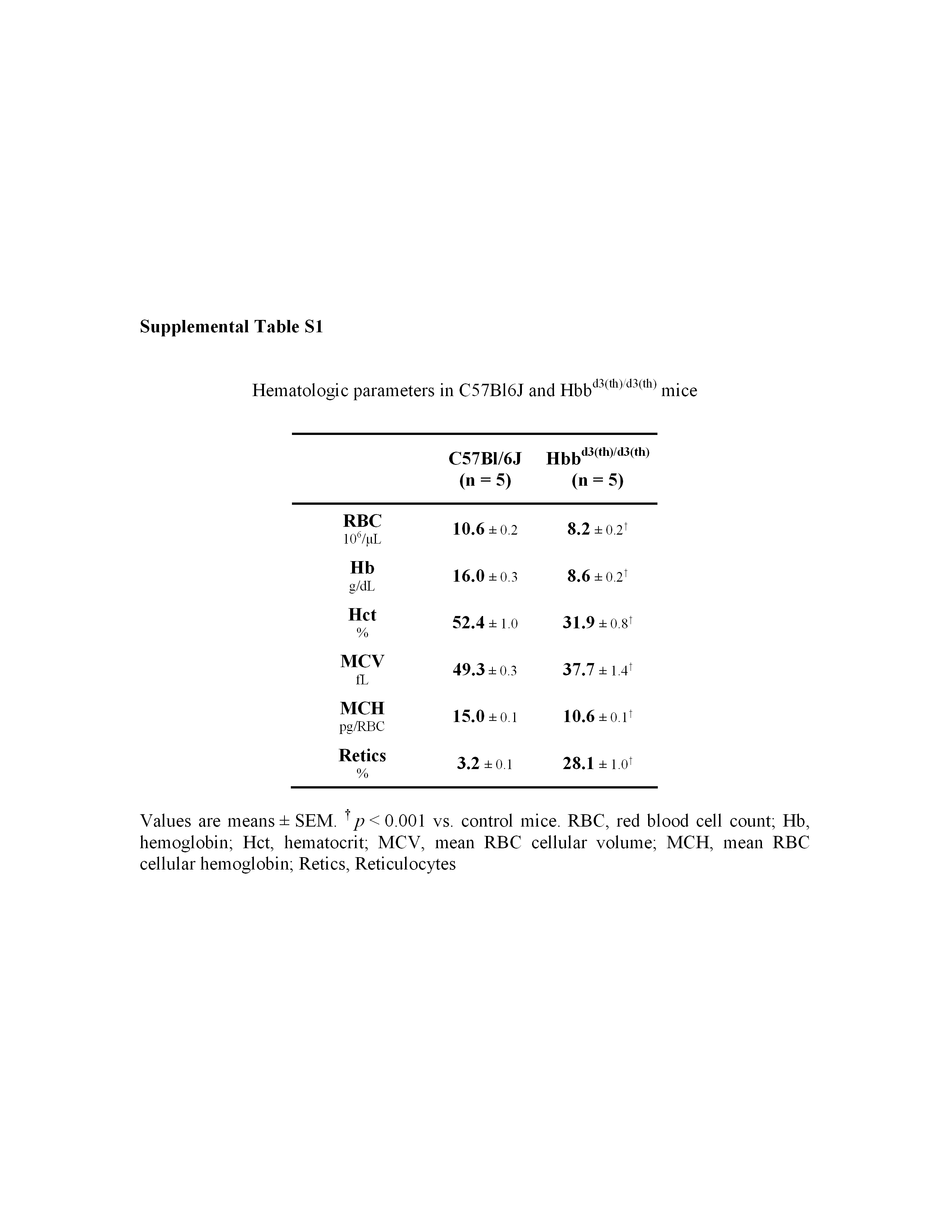

Supplement: Table S1 — Hematologic parameters in C57Bl6J and Hbbd3(th)/d3(th) mice. RBC, red blood cell count; Hb, hemoglobin; Hct, hematocrit; MCV, mean RBC cellular volume; MCH, mean RBC cellular hemoglobin; Retics, Reticulocytes. (TIF) [file pone.0052128.s004.tif]
